# Incidence and impact of hypoattenuated leaflet thickening following aortic valve replacement using a glycerol-preserved bioprosthesis

**DOI:** 10.1016/j.xjon.2023.12.011

**Published:** 2024-01-06

**Authors:** Bastien Poitier, Pierre Dahdah, Margaux Bernardini, Lucas Coroyer, Mohamed Nouar, Ramzi Abi Akar, Alain Bel, David M. Smadja, Leonora Du Puy-Montbrun, Paul Achouh

**Affiliations:** a Cardiac Surgery Department, AP-HP, Georges Pompidou European Hospital, Paris, France; b Hematology Department, AP-HP, Georges Pompidou European Hospital, Paris, France

**Figure:**
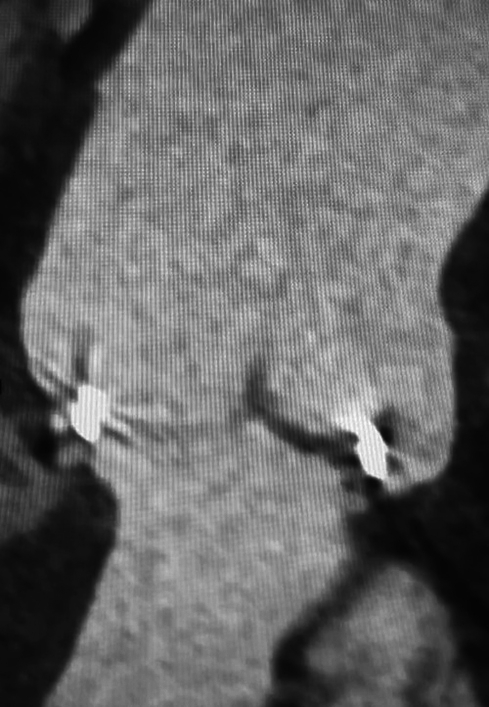
HALT visualized through CT-scan imaging on an INSPIRIS bioprosthesis at the 3-month mark.

**Central Message**

The rare HALT visualized on the RESILIA tissue occurred exclusively in patients under antiplatelet therapy. Post operative anticoagulant strategies could be preferred to enhance valve durability.

Subclinical leaflet thrombosis is characterized by a thin thrombus layer enveloping the leaflets, termed hypoattenuating leaflet thickening (HALT), as visualized through computed tomography (CT) scan imaging, whereas transthoracic echocardiography (TTE) findings may remain normal. The INSPIRIS RESILIA bioprosthesis (Edwards Lifesciences), constructed using RESILIA tissue, is designed to eradicate free aldehydes, a pivotal factor in mitigating tissue calcification. Encouraging 5-year outcomes have been observed.^1^ Nevertheless, the existing literature lacks data on follow-up CT imaging. This study aimed to assess HALT incidence through cardiac CT scan 3 months’ post-surgical aortic valve replacement (SAVR) using this valve.

## Methods

All patients provided informed written consent for future use of their data. In addition, this study was approved by the institutional review board of the French Society of Thoracic and Cardio-Vascular Surgery (no. 2023-10-24_31457, IRB00012919, October 24, 2023).

One hundred thirty-four patients who underwent SAVR at the Georges Pompidou European Hospital with the INSPIRIS RESILIA bioprosthesis from February 6, 2019, to December 31, 2022, were followed-up at the 3-month mark through multiphasic electrocardiogram-gated cardiac CT scan and TTE.

Following SAVR, therapeutic anticoagulation through low-molecular-weight heparin was administered to all patients for 5 days. Subsequently, a singular antiplatelet therapy (SAPT) involving low-dose aspirin was prescribed for 3 months, except for patients who exhibited postoperative atrial fibrillation and were consequently placed on vitamin K antagonists (VKAs) (target international normalized ratio 2-3), as well as individuals with a pre-existing indication for anticoagulation. Should HALT manifest, the choice of anticoagulation was based on the physician's discretion. HALT resolution was assessed after 6 months through a novel cardiac CT scan.

Continuous variables were compared using Mann–Whitney *U* test and categorial variables using the χ^2^ test. All analyses were 2-sided. Statistical analyses and graphs were realized using GraphPad software.

## Results

**Figure 1:**
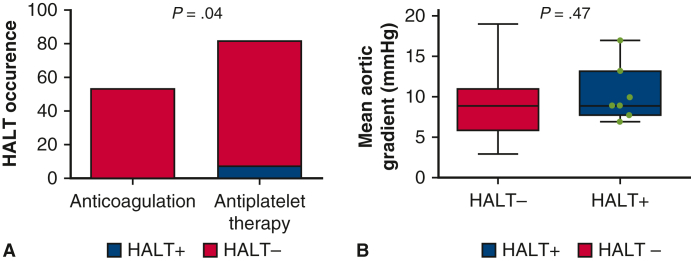
A, HALT occurrence according to antithrombotic treatment. B, Mean aortic gradient in HALT and no-HALT patients. The *lower and upper borders of the box* represent the lower and upper quartiles (25th percentile and 75th percentile). The *middle horizontal line* represents the median. The *lower and upper whiskers* represent the minimum and maximum values. *HALT*, Hypoattenuating leaflet thickening.

The mean age at implantation was 64.2 ± 9.3 years (baseline characteristics are delineated in Table 1). Eighty-one patients (60.4%) were discharged on SAPT. Forty-one patients (30.6%) were discharged on anticoagulants due to pre-existing indication (38 patients on VKA and 3 patients on direct oral anticoagulants [DOACs]), whereas 12 patients (9%) were newly placed on VKAs due to postoperative atrial fibrillation. Two patients placed on VKAs due to pre-existing indication stopped temporarily their treatment due to BARC type 2 bleeds type 2 bleeds during the follow-up. HALT was detected in 7 patients (5.1%), of whom 3 exhibited mild or moderate reduced leaflet motion. None of the patients with HALT reported clinical symptoms or thromboembolic events. Notably, patients treated with SAPT were at a heightened risk of HALT compared with those receiving anticoagulants (7 patients [8.6%] vs 0 [0%], *P* = .04) (Figure 1, *A*). In all patients, TTE revealed a mean aortic gradient of 9.2 ± 3.6 mm Hg, a peak aortic valve velocity of 1.9 ± 0.4 m/s, and an estimated orifice area index of 1.2 ± 0.4 cm^2^/m^2^. No case of hemodynamic valve deterioration was reported. Furthermore, patients with HALT did not present significantly greater mean aortic gradient levels than those without HALT (10.3 ± 3.7 mm Hg vs 9.1 ± 3.6 mm Hg; *P* = .47) (Figure 1, *B*). HALT resolved in 6 patients (85.7%), 5 of whom after treated by DOAC and 1 after continued SAPT. The remaining patient who did not experience HALT resolution was prescribed DOAC. No bleeds occurred in patients treated for HALT.

**Table 1:** Patient baseline characteristics comparison between HALT and no-HALT group

Characteristics	HALT (n = 7)	No HALT (n = 127)	*P* value
Age, y			
Mean ± SD	63.3 ± 8.1	64.3 ± 9.3	.57
Range (min-max)	53.0-78.0	32.0-84.0	
Gender/sex, n (%)			
Female	1 (14.3)	31 (24.4)	.54
Male	6 (85.7)	96 (75.6)	
Tobacco	1 (14.3)	36 (28.3)	.67
Systemic hypertension	4 (57.1)	67 (52.8)	.82
Dyslipidemia	1 (14.3)	42 (33.1)	.30
Diabetes	0 (0)	18 (14.2)	.28
Left ventricular ejection fraction			
Mean ± SD	58.7 ± 11.9	58.9 ± 8.4	.86
Range (min-max)	33.0-69.0	25-71	
EuroSCORE II (%)			
Mean ± SD	2.2 ± 1.3	5.0 ± 7.2	.36
Range (min-max)	0.5-4.6	0.4-48.69	

*HALT*, Hypoattenuating leaflet thickening; *SD*, standard deviation; *EuroSCORE II*, European System for Cardiac Operative Risk Evaluation.

## Discussion

Bioprosthetic heart valve hemocompatibility is acquired during the initial months postsurgery as the pericardium undergoes endothelialization. Throughout this period, bioprosthetic heart valves remain susceptible to thrombosis. The existing literature reports up to 30% of HALT at 1 year after transcatheter aortic valve replacement and 28% after SAVR using multiple valve types.^2^^,^^3^ In our study, we documented SLT in 5.1% of cases at the 3-month mark, a phenomenon exclusively observed in patients under SAPT. Evidence has begun linking SLT to premature structural valve deterioration (SVD). We recently demonstrated heightened calcification due to fibrin deposition on bovine pericardium tissue in a rat model.^4^ The GALILEO trial reported a diminished SLT incidence in patients treated with rivaroxaban versus those undergoing antiplatelet therapy post-transcatheter aortic valve replacement but elevated rates of mortality and bleeding complications.^5^ Importantly, participants in the GALILEO trial were older than our patient cohort. The potential use of different anticoagulants strategies in a younger demographic might offer safety and benefits, particularly in preventing early SVD via inhibition of fibrin deposition. Lastly, the approach to treating SLT with anticoagulants remains nebulous.

In conclusion, the INSPIRIS RESILIA bioprosthesis showed a good hemocompatibility. A more extensive patient cohort and extended follow-up are necessary to corroborate our findings and to prospectively investigate whether HALT is a risk factor for early SVD.

## Conflict of Interest Statement

The authors reported no conflicts of interest.

The *Journal* policy requires editors and reviewers to disclose conflicts of interest and to decline handling or reviewing manuscripts for which they may have a conflict of interest. The editors and reviewers of this article have no conflicts of interest.
